# The pretesting effect under divided attention

**DOI:** 10.1007/s00426-025-02106-3

**Published:** 2025-03-27

**Authors:** Johannes Bartl, Oliver Kliegl, Karl-Heinz T. Bäuml

**Affiliations:** Department of Experimental Psychology, Regensburg University, 93040 Regensburg, Germany

## Abstract

Completing a pretest (e.g., star—?) before receiving correct-answer feedback (e.g., star—*night*) can improve long-term retention of the material compared to material that was initially only studied. The present study examined whether this pretesting effect requires attentional resources during the initial pretest stage and the subsequent feedback stage. Two experiments were conducted in which participants studied word pairs which were either presented in full for 12 s and thus could be studied immediately (study-only trials) or were first only presented with the cue word of a pair and asked to guess the target word for 6 s before the complete pair was shown for another 6 s (pretest trials). Critically, learning occurred either under full attention or under distraction by a secondary task, with the distraction occurring either during the first 6 s or the last 6 s of a trial. While results showed the typical pretesting effect in the absence of any distraction, the effect remained intact when distraction occurred during the first 6 s of a pretest trial, but was eliminated when distraction occurred during the last 6 s. This pattern of results arose when distraction induced material-general (Experiment 1) and when it induced material-specific (Experiment 2) interference. Consistently, additional analyses showed greater recall impairments for pretested pairs when distraction occurred during Stage 2 than during Stage 1, although such impairment was present in both situations. The findings align with theoretical accounts suggesting critical roles of attentional processes for the pretesting effect.

## Introduction

Memory tests not only provide an instrument for assessing a person’s current level of knowledge but are themselves effective learning strategies that can boost memory performance. A particularly striking demonstration of the beneficial effects of testing on memory is the testing effect (for review, see Karpicke, 2017). In a typical testing effect task, participants may, for instance, first study cue-target pairs (star—*night*) before they are either presented with the complete pair again and asked to restudy that pair, or are presented with only the cue word of a pair and asked to retrieve the target word of that pair (star—*?*). A subsequent retention test often shows greater recall of previously tested pairs than of previously restudied pairs (e.g., Kornell et al., 2011). The testing effect is a robust phenomenon which has been shown to arise over a wide variety of learning materials, age groups, and ability levels (for reviews, see Karpicke, 2017; Roediger & Butler, 2011), and has also been observed even in classroom settings with realistic course material (for reviews, see Agarwal et al., 2021; Yang et al., 2021).

Strikingly, beneficial effects of testing can arise even when the test takes place before the to-be-learned material has been exposed (for a review, see Pan & Carpenter, 2023). In a typical task to examine this pretesting effect, participants may be asked to study cue-target pairs that are either presented intact (e.g., star—*night*) and thus can be studied immediately (study-only condition), or of which at first only the cue words are shown for a few seconds (star—*?*) and subjects are asked to guess the target word before the complete pair is revealed (star—*night*; pretest condition). On a later memory test, in which only the cue words are shown and participants are asked to recall the corresponding target words, recall performance is typically enhanced for pairs that were initially pretested compared to pairs that were studied only (e.g., Kornell et al., 2009). The pretesting effect is a robust phenomenon which, over the years, has been found for various types of study materials such as trivia questions, weakly associated word pairs, videos, and text passages, and has been observed in both lab-based studies and educational settings (for reviews, see Chan et al., 2018; Kornell & Vaughn, 2016).

While the effectiveness of pretests as a learning tool thus has been established, it is less clear what types of cognitive processes underlie the pretesting effect. One approach of examining the driving force behind the effect would be to determine which of the two stages of the learning trial in the typical pretesting-effect procedure plays the more critical role for the effect. Stage 1 here refers to the first few seconds of a learning trial during which participants are either asked to guess the target word (pretest condition) or are presented already with the intact cue-target pair (study-only condition); in contrast, Stage 2 refers to the last few seconds of the learning trial during which either the correct-answer feedback is provided (pretest condition) or participants simply continue studying the pair (study-only condition).

One prominent explanation of the pretesting effect is the attentional account, which assumes that Stage 2 of the learning trial is primarily responsible for the pretesting effect (Potts & Shanks, 2014; Zawadzka & Hanczakowski, 2019). Here, the proposal is that pretesting induces greater engagement with the target information once the target is revealed during Stage 2, thus leading to improved recall of the target word on the final test and inducing the pretesting effect. Pretesting may, for instance, increase participants’ curiosity about the correct response, which leads them to allocate more attentional resources for the encoding of the target information during Stage 2. Support for the attentional account comes from prior work showing that participants rated their curiosity about the correct response higher if the rating was given after, rather than before, a guessing attempt had been made during pretesting, suggesting that pretests may indeed raise participants’ curiosity about the correct response (Potts et al., 2019).

Two important alternative explanations of the pretesting effect are the elaboration account and the search-set account. The elaboration account assumes that pretesting activates memory representations which become integrated with the cue-target pair once feedback is provided and facilitate later access to the target information on the final test, thereby creating the pretesting effect (Huelser & Metcalfe, 2012). In contrast, according to the search-set account, pretesting should activate a search set of memory representations that includes also the target information, which should lead to a more efficient encoding of the target information, thus enabling the pretesting effect (e.g., Grimaldi & Karpicke, 2012). Critically, however, neither the elaboration account nor the search-set account posit a clear role for attentional processes and thus do not allow for unambiguous predictions about the effects of divided attention on the pretesting effect (for a more detailed evaluation of the two accounts, see General Discussion section).

This study addresses the role of Stage 1 and Stage 2 of the learning trial for the pretesting effect by examining whether at least one of the two stages requires attentional resources to create the effect. This approach is interesting in its own but also provides a test of the attentional account of the pretesting effect. To investigate the role of attention for memory, researchers often compare participants’ performance in a divided-attention condition, in which a secondary distractor task needs to be performed in parallel to the (primary) memory task, to a full-attention condition, in which participants only engage in the memory task (Craik et al., 1996; Lozito & Mulligan, 2010). A typical finding is that while dividing attention during the initial encoding stage of a memory task can dramatically impair later recall performance (e.g., Anderson et al., 1998), dividing attention during the later memory test often does not result in much recall impairment (e.g., Craik et al., 1996). More recent work using the testing-effect task has shown that divided attention also during the initial (post)test barely affects later recall performance, suggesting that memory retrieval may be largely resilient to distraction from a secondary task (e.g., Buchin & Mulligan, 2017, 2019; for a more thorough discussion, see General Discussion).

Secondary tasks can be distinguished on the basis of whether they induce material-specific interference, which means that the materials of the secondary task and the memory task are of the same type (e.g., words) and thus compete for resources from the same representational system, or material-general interference, which means that the two tasks use material from different categories (e.g., words and numbers), with the two tasks competing for general processing resources only (see Mulligan, 2008, for a review). Material-specific interference often induces stronger detrimental effects on memory than material-general interference (e.g. Fernandes & Moscovitch, 2003). To date, it has not been investigated whether the two types of secondary task modulate the pretesting effect. The only exception is a recent study by Mulligan and Buchin (2024), which used a material-general secondary task in both Stage 1 and Stage 2 of the learning trial and found that the pretesting effect persisted with this type of divided attention. This study is discussed in more detail in the General Discussion section.

### The present study

The goal of the present study was to examine whether the pretesting effect depends on attention-requiring processes during the initial pretest (Stage 1) or the subsequent feedback stage (Stage 2) of the learning trial. To this end, we conducted two experiments in both of which word pairs were either presented in full for 12 s and thus could be studied immediately (study-only trials) or were first presented with the cue word of a pair only and subjects were asked to guess the target word (star—*?*) before the complete pair was shown for another 6 s (pretest trials). For one half of the trials, learning took place with full attention, whereas for the remaining half of the trials, learning took place under divided attention, with distraction created by a secondary task. In this secondary task, which was performed in parallel to the learning task in both experiments, participants either were asked to classify numbers as odd or even (Experiment 1), which was assumed to induce material-general interference, or to classify nouns as either ‘man-made’ or ‘coming from nature’ (Experiment 2), which was assumed to induce material-specific interference—and thus higher levels of competition—for the memory task. The secondary task was applied during either Stage 1 (Experiments 1a and 2a) or Stage 2 (Experiments 1b and 2b) of the learning task. In a later retention test, subjects’ memory was assessed for all word pairs by providing a pair’s cue word and asking subjects to provide the correct target word.

In both experiments, learning under full attention was assumed to create a reliable pretesting effect, with greater recall for pretest than study-only pairs on the final test, replicating prior work (e.g., Kornell et al., 2009). However, it was less clear how divided attention during either Stage 1 or Stage 2 would affect the size of the pretesting effect. If the pretesting effect is primarily induced by attention-requiring processes operating during Stage 2—as suggested by the attentional account of the pretesting effect—then engaging in a secondary task during Stage 1 should barely affect the pretesting effect, whereas distraction during Stage 2 should reduce the effect. Furthermore, based on the attentional account and on prior pretesting effect studies showing that manipulations which increase correct recall often reduce commission errors (e.g., Kliegl et al., 2023, 2024a), pretesting may only reduce commission errors when the secondary task occurs during Stage 1, but not during Stage 2. The results of the present study thus will be of high relevance for a better understanding of the role of attention-requiring cognitive processes for the pretesting effect.

## Experiments 1a and 1b

Apart from a small procedural difference, the methods of Experiment 1a and Experiment 1b are identical and are therefore reported together.

### Method

#### Ethical considerations

All reported studies were conducted in accordance with the provisions of the Declaration of Helsinki of the World Medical Association.

#### Participants

To determine sample size in all experiments, a power analysis was conducted using G*Power (version 3.1.9.2; Faul et al., 2009). Based on the meta-analytic effect size estimate of the pretesting effect (Hedge’s *g* = 0.44; Boustani & Shanks, 2022), a two-sample t test used to determine if two population means are equal revealed that 43 subjects were required overall when alpha was set to 0.05 and beta was set to 0.20. The actual sample size was somewhat higher than recommended, with 52 students each taking part in Experiment 1a (mean age = 25.3 years; SD = 4.1 years; 35 female, 17 male, 0 diverse) and Experiment 1b (mean age = 25.1 years; SD = 4.0 years; 39 female, 13 male, 0 diverse). All subjects spoke German as their native language. We did not assess whether participants had a neurological or psychiatric diagnosis. All subjects gave their spoken informed consent and received either course credit or a compensatory amount of money for their participation.

#### Material

Sixty weakly associated word pairs (e.g., blood—*plasma*; train—*plane*) drawn from the Nelson et al. (1998) norms were used as study materials. The forward association strength of the pairs was 0.052 on average (ranging from 0.047 to 0.067), meaning that, when presented with the cue word of a pair, subjects produced the target word as their first response in approximately 5% of all cases. All items were translated into German. For each subject, the 60 cue-target pairs were randomly divided into two subsets consisting of 30 pairs each, with each subset being allocated to one of the two experimental blocks. For both blocks, 15 of the 30 pairs were randomly assigned to pretest trials and the remaining 15 pairs were assigned to study-only trials.

#### Design and procedure

**Fig. 1:**
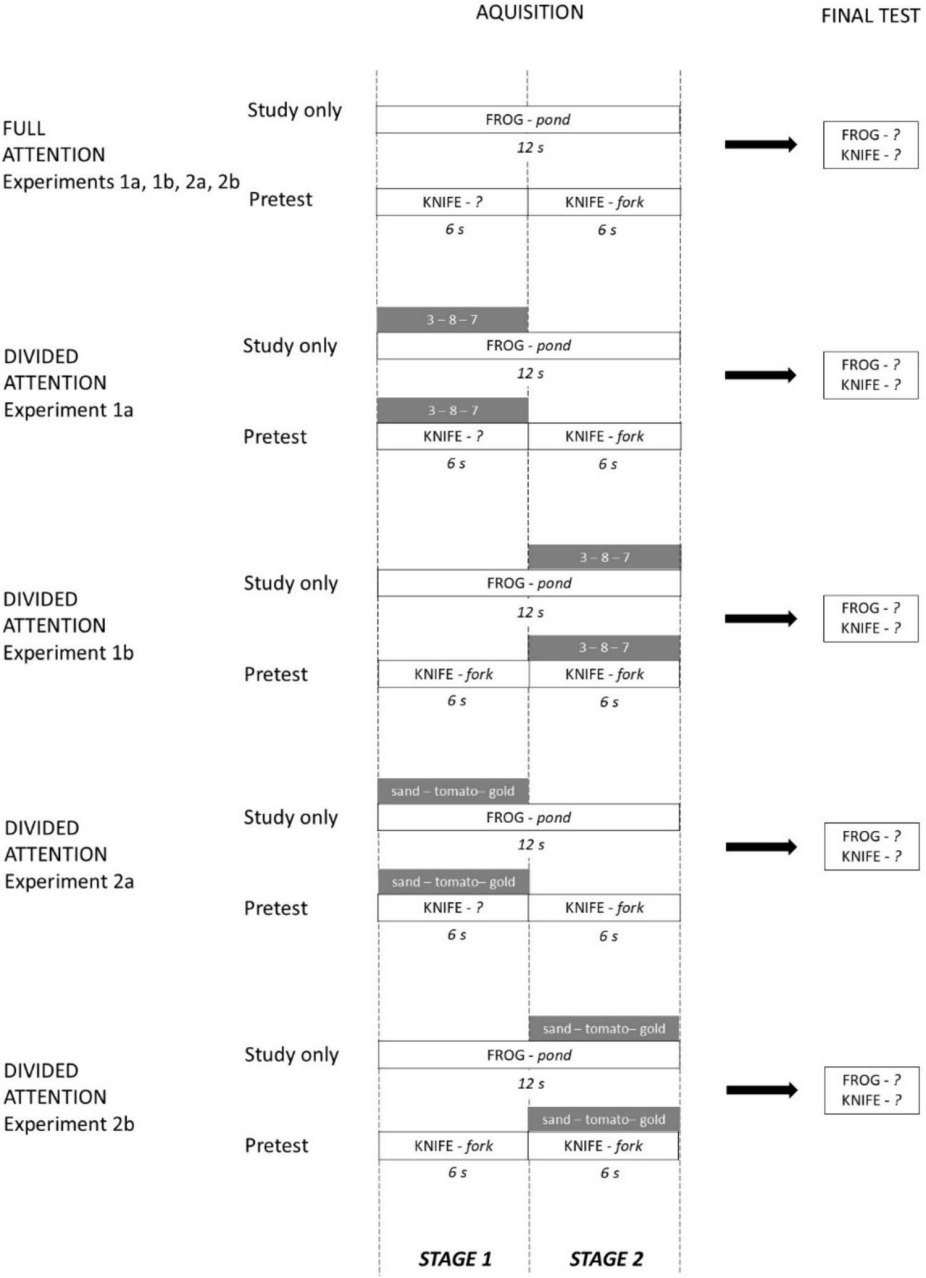
Procedure of Experiments 1a, 1b, 2a, and 2b: In all four experiments, participants studied word pairs which were either presented in full for 12 s and thus could be learned immediately (study-only trials) or were first only presented with the cue word of a pair and asked to guess the target word for 6 s before the complete pair was shown for another 6 s (pretest trials). Learning occurred either under full attention or under distraction from a secondary task during Stage 1, i.e., the first 6 s of the trial (Experiments 1a and 2a) or Stage 2, i.e., the last 6 s of a trial (Experiments 1b and 2b). In Experiments 1a and 1b, subjects were presented with three digits in succession during this secondary task and asked to indicate for each digit whether it was even or odd. On a subsequent final test, participants were asked to recall all initially studied target words. All procedural details of Experiments 2a and 2b were identical to Experiments 1a and 1b, with the critical exception that nouns were presented instead of digits in the secondary task, and participants had to indicate whether a given noun referred to a natural or man-made object

Experiments 1a and 1b were conducted online via individual meetings using the videotelephony software program Zoom (Zoom Video Communications). Both experiments consisted of an acquisition phase and a final test phase, which are illustrated in Fig. 1. The acquisition phase consisted of two different blocks with 30 learning trials each. Each learning trial was split into two stages: Stage 1 comprised the first 6 s of the learning trial and Stage 2 included the second 6 s of the learning trial. Half of the cue words of each block were presented together with their target word (e.g., blood—*plasma*) in Stage 1 and thus could be studied immediately (study-only condition), whereas for the other half of the word pairs in Stage 1 only the cue word (e.g., train—*?*) was presented for 6 s and participants had to guess the corresponding target word (pretest condition). Guesses were made verbally and recorded in writing by the experimenter. All items in this (primary) memory task were presented on the lower half of the screen. During Stage 2 of the learning trial, which followed Stage 1 without any interruption, the intact word pair (e.g., blood—*plasma*, train—*plane*) was shown for 6 s in both conditions. While, in study-only trials, Stages 1 and 2 simply appeared as a single 12-s period in which the complete cue-target pair could be studied from the start, in pretest trials, Stage 2 appeared as a 6-s feedback period that followed the preceding pretest period of Stage 1. All word pairs were presented in a randomized order for each participant and displayed in the lower half of the screen underneath a fixation cross.

For each participant, one learning block was under full attention (as described above) and the remaining block was under divided attention. While half of participants first took part in the full-attention block and subsequently in the divided-attention block, for the remaining half of participants, the order of blocks was reversed. The divided attention block was identical to the full-attention block, with the only difference that participants were asked to engage in a secondary task. The secondary task was applied during either Stage 1 (Experiment 1a) or Stage 2 (Experiment 1b) of the learning trial, and subjects thus had to complete it in parallel to the (primary) memory task. Participants were told at the beginning of the divided-attention block that the (secondary) digit-classification task was equally important as the (primary) memory task. For the secondary task, three digits (1–9, excluding 5) were randomly drawn and presented sequentially on the top half of the screen for 2 s each. Participants were asked to categorize these digits as fast and correctly as possible as either an even number, by pressing the ‘g’ button on their keyboard, or as an odd number, by pressing the ‘u’ button. At the end of each learning trial, participants received feedback about their accuracy in the secondary task (e.g., ‘67 percent of your answers were correct’ if two out of three responses were correct) for 1.5 s before the start of the subsequent trial.

After the acquisition phase, all participants were asked to play an online version of the video game Tetris (https://www.geo.de/geolino/spiele/13349-rtkl-onlinespiel-tetris) for 5 min before the final test was administered. On this test, the cue words of all 60 word pairs were presented for 10 s each in randomized order and participants were asked to name the corresponding target word (e.g., blood—*?*). All answers during the pretesting and the final test were provided verbally to the experimenter. No feedback was given during the final test. Experiments 1a and 1b thus consisted of a 2 × 2 design with the within-subjects factors type of practice (study only vs. pretest) and level of attention (full attention vs. divided attention).

#### Transparency and openness

All study materials and data have been made publicly available on the Open Science Framework and can be found at https://osf.io/nxdzv/.

### Results of Experiment 1a

#### Initial pretest

On the initial pretest, the proportion of correctly guessed target words did not differ significantly between pretest trials under full attention and pretest trials under divided attention (4.9% vs. 4.4%), *t*(51) = 0.43, *p* = 0.67, Cohen’s *d* = 0.06. Since the effect of erroneous guesses on subsequent memory was of main interest, all items that were correctly guessed during the pretest were removed from further analyses.

#### Final test

##### Correct recall

**Fig. 2:**
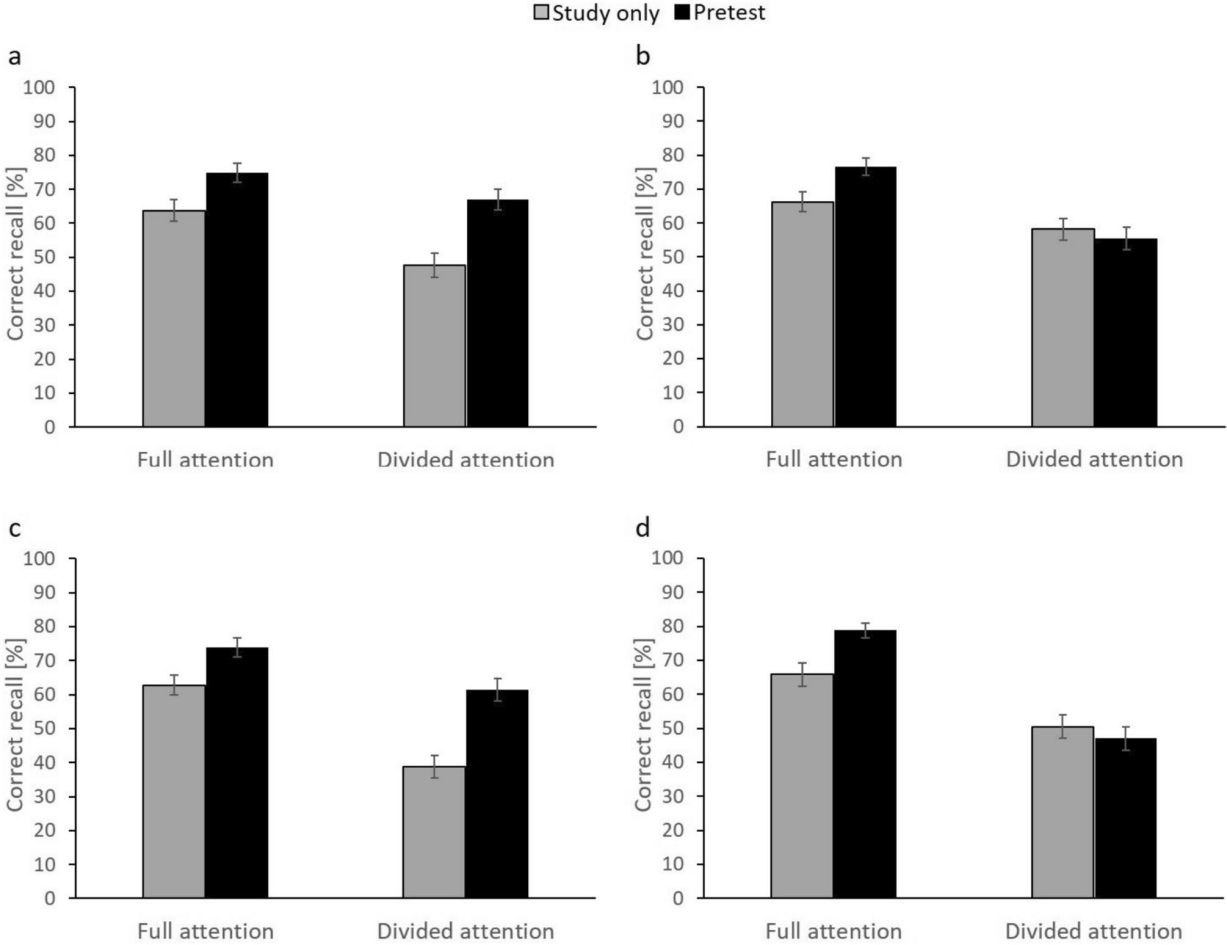
**a** Results of Experiment 1a. Recall performance on the final test (in %) as a function of level of attention (full attention vs. divided attention) and type of practice (study only vs. pretest). **b** Results of Experiment 1b. Recall performance on the final test (in %) as a function of level of attention (full attention vs. divided attention) and type of practice (study only vs. pretest). **c** Results of Experiment 2a. Recall performance on the final test (in %) as a function of level of attention (full attention vs. divided attention) and type of practice (study only vs. pretest). **d** Results of Experiment 2b. Recall performance on the final test (in %) as a function of level of attention (full attention vs. divided attention) and type of practice (study only vs. pretest). Error bars reflect standard errors

Figure 2a shows the percentage of correctly recalled target words on the final test as a function of TYPE OF PRACTICE (study only vs. pretest) and LEVEL OF ATTENTION (full attention vs. divided attention). A 2 × 2 analysis of variance (ANOVA) of the two factors on correct-recall performance revealed significant main effects of TYPE OF PRACTICE, *F*(1, 51) = 62.79, MSE = 133.76, *p* < 0.001, *η*_*p*_^2^ = 0.55, and LEVEL OF ATTENTION, *F*(1, 51) = 23.85, MSE = 133.76*, p* < 133.76*, η*_*p*_^2^ = 0.32, reflecting overall higher recall rates for pretest pairs than for study-only pairs (70.9% vs. 55.6%) and for pairs studied under full than divided attention (69.3% vs. 57.3%). These main effects were qualified by a statistically significant interaction between the two factors, *F*(1, 51) = 6.76, MSE = 133.76, *p* = 0.012, *η*_*p*_^2^ = 0.12. Consistently, pairwise comparisons revealed that although final-test recall was better in the pretest condition than the study-only condition, both for word pairs under full attention (74.9% vs. 63.7%), *t*(51) = 5.60, *p* < 0.001, Cohen’s *d* = 0.78, and under divided attention (67.1% vs. 47.6%), *t*(51) = 6.62, *p* < 0.001, Cohen’s *d* = 0.92, the size of the pretesting effect increased from 11.1% under full attention to 19.5% under divided attention.

##### Restricted analysis

In the divided-attention condition, subjects performed the (secondary) digit-classification task while simultaneously engaging in the (primary) memory task. A pairwise comparison revealed that performance on the secondary task was better in the study-only condition than in the pretest condition (89.9% vs. 78.5%), *t*(51) = 6.31, *p* < 0.001, Cohen’s *d* = 0.88. To rule out the possibility that the interaction between TYPE OF PRACTICE and LEVEL OF ATTENTION reported above arose only due to the fact that more attentional resources were allocated to the (primary) memory task in the pretest condition than in the study-only condition, a restricted analysis was conducted. In this analysis, only trials in which participants responded 100% correctly in the secondary task were included (for a similar proceeding, see Buchin & Mulligan, 2017).^1^ Using this data set together with the data set including the full-attention trials (which remained identical as there was no secondary task), the same 2 × 2 ANOVA was performed. Like the unrestricted analysis, results showed a significant interaction of the two factors, *F*(1,50) = 5.21, MSE = 245.50, *p* = 0.027, *η*_*p*_^2^ = 0.09, indicating that even when secondary-task performance was equated for the study-only and pretest conditions, the pretesting effect was still larger under divided attention than under full attention.

##### Intrusions

All commission errors that participants produced during the final test were counted as intrusions. A 2 × 2 ANOVA with the factors TYPE OF PRACTICE (study only vs. pretest) and LEVEL OF ATTENTION (full attention vs. divided attention) on number of intrusions revealed significant main effects of TYPE OF PRACTICE, *F*(1,51) = 33.31, MSE = 2.33, *p* < 0.001, *η*_*p*_^2^ = 0.40*,* and LEVEL OF ATTENTION, *F*(1,51) = 12.24, MSE = 2.33, *p* < 0.001,* η*_*p*_^2^ = 0.19, reflecting that, overall, pretest trials led to a lower number of intrusions than study-only trials (2.7 vs. 4.4 intrusions) and intrusions were lower for trials under full attention than for trials under divided attention (3.1 vs. 4.0 intrusions). There was also a significant interaction between both factors, *F*(1,51) = 6.47, MSE = 2.33, *p* = 0.014, *η*_*p*_^2^ = 0.11. Consistently, while pairwise comparisons revealed that pretest trials led to fewer intrusions than study-only trials under both full attention, *t*(51) = 3.68, *p* < 0.001, Cohen’s *d* = 0.51, and under divided attention, *t*(51) = 5.44, *p* < 0.001, Cohen’s *d* = 0.76, the magnitude of this benefit of pretesting increased from 1.2 (2.5 vs. 3.7 intrusions) under full attention to 2.2 (2.9 vs. 5.1 intrusions) under divided attention (see Table 1).

**Table 1:** Mean number of intrusions (standard error of the mean) produced on the final test as a function of LEVEL OF ATTENTION and TYPE OF PRACTICE for Experiments 1 and 2

Experiment	Full attention		Divided attention	
	Study	Pretest	Study	Pretest
Experiment 1a	3.65 (0.46)	2.54 (0.35)	5.12 (0.56)	2.92 (0.34)
Experiment 1b	3.25 (0.39)	1.96 (0.36)	4.00 (0.43)	3.77 (0.45)
Experiment 2a	3.62 (0.38)	2.21 (0.31)	6.33 (0.53)	3.83 (0.43)
Experiment 2b	3.46 (0.45)	2.04 (0.28)	5.04 (0.48)	5.12 (0.50)

### Results of Experiment 1b

#### Initial pretest

On the pretest, there was again no significant difference in the proportion of correctly guessed target words between trials under full attention and trials under divided attention (5.1% vs. 4.1%), *t*(51) = 1.16, *p* = 0.25, Cohen’s *d* = 0.16. Since the effect of erroneous guesses on subsequent memory was of main interest, all items that were correctly guessed during the pretest were removed from further analyses.

#### Final test

##### Correct recall

Figure 2b shows the percentage of correctly recalled target words on the final test as a function of TYPE OF PRACTICE (study only vs. pretest) and LEVEL OF ATTENTION (full attention vs. divided attention). A 2 × 2 ANOVA of the two factors on correct-recall performance revealed a significant main effect of LEVEL OF ATTENTION, *F*(1,51) = 36.66, MSE = 122.17, *p* < 0.001, *η*_*p*_^2^ = 0.42, reflecting overall higher recall rates for pairs studied under full than divided attention (71.4% vs. 56.9%), but no main effect of TYPE OF PRACTICE, *F*(1,51) = 3.40, MSE = 122.17, *p* = 0.071, *η*_*p*_^2^ = 0.06. Most important, there was a statistically significant interaction between the two factors, *F*(1,51) = 18.02, MSE = 122.17*, p* < 0.001*, η*_*p*_^2^ = 0.26. Consistently, pairwise comparisons revealed that, relative to the study-only condition, recall in the pretest condition was significantly improved for word pairs under full attention (66.3% vs. 76.6%), *t*(51) = 4.45, *p* < 0.001, Cohen’s *d* = 0.62, as reflected in a pretesting effect of 10.3%, but did not differ between conditions under divided attention (58.2% vs. 55.5%), *t*(51) = 0.97, *p* = 0.34, Cohen’s *d* = 0.14.

##### Restricted analysis

As in Experiment 1a, performance in the digit classification task was superior in the study-only condition relative to the pretest condition (87.7% vs. 76.0%), *t*(51) = 7.35, *p* < 0.001, Cohen’s *d* = 1.02. Like the unrestricted analysis, results of the restricted 2 × 2 ANOVA—including only trials of the divided-attention condition in which participants responded 100% correctly in the secondary task—showed a significant interaction of the two factors, *F*(1,50) = 8.97, MSE = 174.39, *p* = 0.004, *η*_*p*_^2^ = 0.15, thus indicating a significant pretesting effect under full attention only.

##### Intrusions

A 2 × 2 ANOVA with the factors TYPE OF PRACTICE (study only vs. pretest) and LEVEL OF ATTENTION (full attention vs. divided attention) on number of intrusions revealed significant main effects of TYPE OF PRACTICE, *F*(1,51) = 10.61, MSE = 1.94, *p* = 0.002, *η*_*p*_^2^ = 0.17, and LEVEL OF ATTENTION, *F*(1,51) = 27.24, MSE = 1.94, *p* < 0.001, *η*_*p*_^2^ = 0.35, reflecting that, overall, pretest trials led to a lower number of intrusions than study-only trials (2.9 vs. 3.6 intrusions) and number of intrusions was lower for trials under full attention than for trials under divided attention (2.6 vs. 3.9 intrusions). There was also a significant interaction between factors, *F*(1,51) = 7.51, MSE = 1.94*, p* = 0.008*, η*_*p*_^2^ = 0.13. In fact, while pairwise comparisons revealed that pretest trials led to fewer intrusions than study-only trials under full attention (2.0 vs. 3.3 intrusions), *t*(51) = 4.49, *p* < 0.001, Cohen’s *d* = 0.62, this benefit of pretesting was absent under divided attention (3.8 vs. 4.0 intrusions), *t*(51) = 0.73, *p* = 0.47, Cohen’s *d* = 0.10 (see Table 1).

### Additional analysis

Final-test recall in the study-only condition was 10.6% lower when the secondary task took place during Stage 1 of the learning trial (Experiment 1a) than when the secondary task occurred during Stage 2 of the learning trial (Experiment 1b). Consequently, the observed pattern of results of Experiment 1a (i.e., an increased pretesting effect under divided than full attention) and Experiment 1b (i.e., an eliminated pretesting effect under divided than full attention) might be attributed at least partially to such variations in the (study-only) baseline condition. To rule out this possibility, we conducted an additional analysis which included only the pooled data of the pretest conditions of Experiments 1a and 1b, excluding the data of the study-only conditions.

A 2 × 2 ANOVA with the factors LEVEL OF ATTENTION (full attention vs. divided attention) and POSITION OF SECONDARY TASK (Stage 1 vs. Stage 2) revealed a significant main effect of LEVEL OF ATTENTION, *F*(1,102) = 51.98, MSE = 208.99, *p* < 0.001, *η*_*p*_^2^ = 0.34, reflecting overall higher recall rates for the full-attention condition than the divided-attention condition (75.7% vs. 61.3%), but no main effect of position of secondary task, *F*(1,102) = 1.77, MSE = 714.71, *p* = 0.19, *η*_*p*_^2^ = 0.02. Critically, there was a significant interaction between the two factors, *F*(1,102) = 10.96, MSE = 208.99, *p* < 0.001, *η*_*p*_^2^ = 0.10. Specifically, while recall was impaired under divided attention, relative to full attention, both when the secondary task occurred in Stage 1 and in Stage 2, all *p*s ≤ 0.004, this difference between the divided-attention and full-attention conditions was greater when the secondary task took place during Stage 2 than during Stage 1 (21.1% vs. 7.8%). The findings of the additional analysis thus converge with the findings from the earlier analyses that included the study-only condition, by suggesting that applying the secondary task during Stage 2 impairs recall of pretested information to a greater degree than applying the secondary task during Stage 1.

### Discussion

The results of Experiments 1a and 1b both showed a typical pretesting effect under full attention, as reflected in enhanced recall on the final test for pretested material, relative to material that was studied only. More important, the results revealed that engaging in a secondary task during learning can eliminate the pretesting effect, but only when the task was applied during Stage 2 of the learning trial (Experiment 1b) and not when the task was applied during Stage 1 (Experiment 1a). The results of Experiment 1a even indicated that the pretesting effect was greater in size under divided than full attention, which was due to the fact that recall performance in the study-only condition was more impaired by divided attention than the pretest condition. For both Experiments 1a and 1b, this pattern of results arose not only when all items were included in the analysis, but also when the analysis was limited to trials for which all responses were correct on the secondary task. For Experiment 1a, this restricted analysis demonstrates that the increased pretesting effect under divided attention did not simply arise because participants strategically allocated more attentional resources to the (primary) memory task in pretest trials than study-only trials.

Furthermore, the analysis of intrusion data revealed a higher number of intrusions on the final test on study-only trials than pretest trials when learning was under full attention, thus replicating prior work (e.g., Kliegl et al., 2024a, 2024b). Under divided attention, pretesting only reduced the number of intrusions when the secondary task occurred during Stage 1, but not during Stage 2, suggesting a pattern of results that is complementary to the correct recall data. Finally, an additional analysis, which analyzed only the pooled data of Experiments 1a and 1b of the pretest condition, showed that compared to learning under full attention, correct recall on the final test was impaired both when the secondary task occurred during Stage 1 and when it occurred during Stage 2. The impairment was, however, much more pronounced when subjects were distracted during Stage 2 than during Stage 1, which suggests that Stage 2 in particular may demand attentional resources that are critical for the pretesting effect, and converges with the results above based on both pretest and study-only trials.

The goal of Experiment 2 was to replicate the results of Experiment 1 conceptually. Experiment 2 differed from Experiment 1 only in the type of secondary task that was applied during learning, i.e., a word classification task instead of a digit classification task. This task was used to investigate whether the results of Experiment 1 would generalize to the domain of material-specific interference, in which the learning task and secondary task compete for resources from the same representational system. Since material-specific interference should induce an even higher level of distraction than material-general interference, it remains open whether, under such conditions, divided attention can lead to the same pattern of results as in Experiment 1.

## Experiments 2a and 2b

Except for a minor procedural difference, the methods of Experiment 2a and Experiment 2b were identical. Therefore, the methods for these experiments are presented together.

### Method

#### Participants

Following the power analysis reported in Experiment 1, 52 subjects each took part in Experiment 2a (mean age = 25.3 years; SD = 4.0 years; 35 female, 17 male, 0 diverse) and Experiment 2b (mean age = 24.5 years; SD = 3.5 years; 35 female,

17 male, 0 diverse). All participants spoke German as their native language and gave their spoken informed consent. In return for their participation, all subject received either course credit or compensatory amount of money.

#### Material, design and procedure

Experiments 2a and 2b were identical to Experiments 1a and 1b, with the only exception that a word classification task was used as the secondary task instead of a digit classification task (see Fig. 1). Ninety nouns were used for this task, half of which could be clearly classified as ‘man-made’ (e.g., *clock*, *stove*, *canoe*) and the other half as ‘coming from nature’ (e.g., *tomato*, *butterfly*, *pond*). Like in Experiment 1, the secondary task was applied during either Stage 1 (Experiment 2a) or Stage 2 (Experiment 2b) of the learning trial. For each trial, three nouns were randomly drawn and presented sequentially on the top half of the screen for 2 s each, and participants were asked to categorize them as fast and correctly as possible as man-made by pressing the ‘m’ button on their keyboard or as coming from nature by pressing the ‘n’ button. Overall, man-made and natural items were presented the same number of times at each position of a secondary task trial.

### Results of Experiment 2a

#### Initial pretest

On the pretest, the proportion of correctly guessed target words did not differ significantly between trials under full attention and trials under divided attention (2.8% vs. 4.4%), *t*(51) = 1.63, *p* = 0.11, Cohen’s *d* = 0.23. Since the effect of erroneous guesses on subsequent memory was of main interest, all items that were correctly guessed during the pretest were removed from further analyses.

#### Final test

##### Correct recall

Figure 2c shows the percentage of correctly recalled target words on the final test as a function of TYPE OF PRACTICE (study only vs. pretest) and LEVEL OF ATTENTION (full attention vs. divided attention). A 2 × 2 ANOVA of the two factors on correct-recall performance revealed significant main effects of TYPE OF PRACTICE, *F*(1,51) = 52.84, MSE = 211.29, *p* < 0.001, *η*_*p*_^2^ = 0.51, and LEVEL OF ATTENTION, *F*(1,51) = 63.47, MSE = 211.29, *p* < 0.001, *η*_*p*_^2^ = 0.55, reflecting overall higher recall rates for pretest pairs than for study-only pairs (67.6% vs. 50.8%) and for pairs studied under full attention than for pairs studied under divided attention (68.3% vs. 50.1%). These main effects were qualified by a statistically significant interaction between the two factors, *F*(1,51) = 8.50, MSE = 211.29,* p* = 0.005,* η*_*p*_^2^ = 0.14. Consistently, pairwise comparisons revealed that although recall was better in the pretest condition than in the study-only condition, both for word pairs under full attention (73.8% vs. 62.8%), *t*(51) = 4.03, *p* < 0.001, Cohen’s *d* = 0.56, and under divided attention (61.5% vs. 38.7%), *t*(51) = 6.72, *p* < 0.001, Cohen’s *d* = 0.93, the size of the pretesting effect increased from 11.0% under full attention to 22.7% under divided attention.

##### Restricted analysis

Like in the prior experiments, a pairwise comparison revealed that secondary-task performance was better in the study-only condition than in the pretest condition (81.8% vs. 65.0%), *t*(51) = 10.99, *p* < 0.001, Cohen’s *d* = 1.52. Like the unrestricted analysis, results of the restricted 2 × 2 ANOVA—including only trials of the divided-attention condition in which participants responded 100% correctly in the secondary task—showed a significant interaction of the two factors, *F*(1,50) = 5.52, MSE = 430.08, *p* = 0.023, *η*_*p*_^2^ = 0.10, indicating that even when secondary-task performance was equated for the study-only and pretest conditions, the pretesting effect was larger under divided attention than under full attention.

##### Intrusions

A 2 × 2 ANOVA with the factors TYPE OF PRACTICE (study only vs. pretest) and LEVEL OF ATTENTION (full attention vs. divided attention) on number of intrusions revealed significant main effects of TYPE OF PRACTICE, *F*(1,51) = 50.24, MSE = 2.46*, p* < 0.001*, η*_*p*_^2^ = 0.50, and LEVEL OF ATTENTION, *F*(1,51) = 62.42, MSE = 2.46, *p* < 0.001, *η*_*p*_^2^ = 0.55, reflecting that, overall, pretest trials led to a lower number of intrusions than study-only trials (3.0 vs. 5.0 intrusions) and number of intrusions was lower for trials under full attention than for trials under divided attention (2.9 vs. 5.1 intrusions). There was also a significant interaction between factors, *F*(1,51) = 6.34, MSE = 2.46, *p* = 0.015, *η*_*p*_^2^ = 0.11. In fact, while pairwise comparisons revealed that pretest trials led to fewer intrusions than study-only trials under both full attention, *t*(51) = 4.43, *p* < 0.001, Cohen’s *d* = 0.61, and divided attention, *t*(51) = 6.54, *p* < 0.001, Cohen’s *d* = 0.91, the magnitude of this benefit of pretesting increased from 1.4 (2.2 vs. 3.6 intrusions) under full attention to 2.5 (3.8 vs. 6.3 intrusions) under divided attention (see Table 1).

### Results of Experiment 2b

#### Initial pretest

On the pretest, there was again no significant difference in the proportion of correctly guessed target words between trials under full attention and trials under divided attention (5.6% vs. 5.9%), *t*(51) = 0.22, *p* = 0.83, Cohen’s *d* = 0.03. Since the effect of erroneous guesses on subsequent memory was of main interest, all items that were correctly guessed during the pretest were removed from further analyses.

#### Final test

##### Correct recall

Figure 2d shows the percentage of correctly recalled target words on the final test as a function of TYPE OF PRACTICE (study only vs. pretest) and LEVEL OF ATTENTION (full attention vs. divided attention). A 2 × 2 ANOVA of the two factors on correct-recall performance revealed significant main effects of TYPE OF PRACTICE, *F*(1,51) = 4.42, MSE = 147.35, *p* = 0.04, *η*_*p*_^2^ = 0.08, and LEVEL OF ATTENTION, *F*(1,51) = 97.70, MSE = 1478.35, *p* < 0.001*, η*_*p*_^2^ = 0.66, reflecting overall higher recall rates for pretested pairs than for studied pairs (62.9% vs. 58.2%) and for pairs studied under full attention than for pairs studied under divided attention (72.4% vs. 48.8%). These main effects were qualified by a statistically significant interaction between the two factors, *F*(1,51) = 23.79, MSE = 147.35, *p* < 0.001, *η*_*p*_^2^ = 0.32. Consistently, pairwise comparisons revealed that, relative to the study-only condition, recall in the pretest condition was improved for word pairs under full attention (65.9% vs. 78.8%), *t*(51) = 4.51, *p* < 0.001, Cohen’s *d* = 0.63, reflecting a pretesting effect of 12.9%, but did not differ between conditions under divided attention (50.5% vs. 47.0%), *t*(51) = 1.28, *p* = 0.21, Cohen’s *d* = 0.18.

##### Restricted analysis

Like in the prior experiments, performance in the secondary task was better in the study-only condition than in the pretest condition (84.4% vs. 71.2%), *t*(51) = 7.38, *p* < 0.001, Cohen’s *d* = 1.02. Like the unrestricted analysis, results of the restricted 2 × 2 ANOVA—including only trials of the divided-attention condition in which participants responded 100% correctly in the secondary task—showed a significant interaction of the two factors, *F*(1,47) = 7.13, MSE = 205.97, *p* = 0.01, *η*_*p*_^2^ = 0.13, thus indicating a reliable pretesting effect under full attention only.

##### Intrusions

A 2 × 2 ANOVA with the factors TYPE OF PRACTICE (study only vs. pretest) and LEVEL OF ATTENTION (full attention vs. divided attention) on number of intrusions revealed significant main effects of TYPE OF PRACTICE, *F*(1,51) = 7.66, MSE = 2.81, *p* = 0.008, *η*_*p*_^2^ = 0.13, and LEVEL OF ATTENTION, *F*(1,51) = 63.55, MSE = 2.81, *p* < 0.001, *η*_*p*_^2^ = 0.56, reflecting that, overall, pretest trials led to a lower number of intrusions than study-only trials (3.6 vs. 4.3 intrusions) and number of intrusions was lower for trials under full attention than for trials under divided attention (2.8 vs. 5.1 intrusions). There was also a significant interaction between factors, *F*(1,51) = 10.41, MSE = 0.281, *p* = 0.002, *η*_*p*_^2^ = 0.17. In fact, while pairwise comparisons revealed that pretest trials led to fewer intrusions than study-only trials under full attention (2.0 vs. 3.5 intrusions), *t*(51) = 3.94, *p* < 0.001, Cohen’s *d* = 0.55, this benefit of pretesting was absent under divided attention (5.1 vs. 5.0 intrusions), *t*(51) = 0.25, *p* = 0.81, Cohen’s *d* = 0.03 (see Table 1).

### Additional analysis

**Fig. 3:**
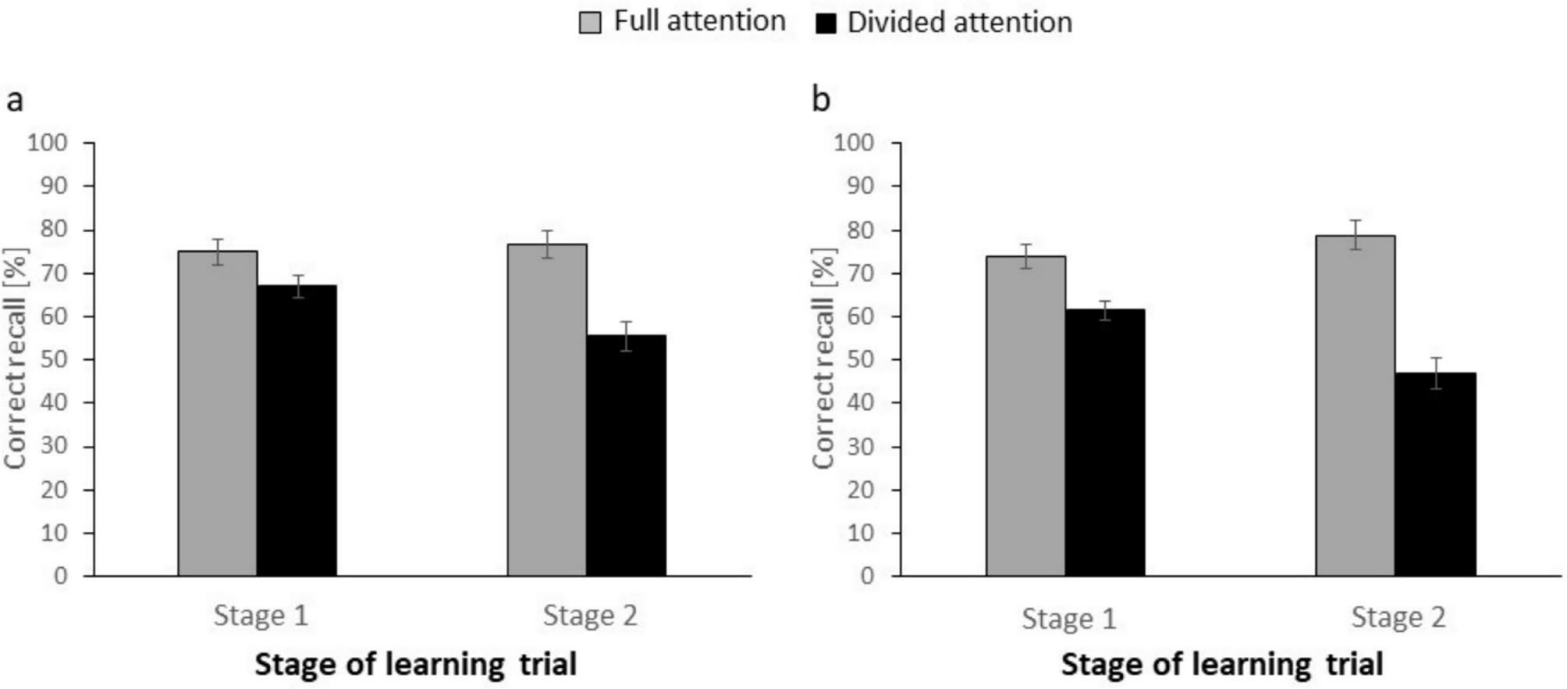
**a** Results of Experiment 1. Recall performance on the final test (in %) as a function of position of secondary task (Stage 1 vs. Stage 2) and level of attention (full attention vs. divided attention). **b** Results of Experiment 2. Recall performance on the final test (in %) as a function of position of secondary task (Stage 1 vs. Stage 2) and level of attention (full attention vs. divided attention). Error bars reflect standard errors

Final-test recall was 11.8% lower when the secondary task took place during Stage. 1 of the learning trial (Experiment 2a) than when the secondary task occurred during Stage 2 of the learning trial (Experiment 2b), which is similar to the results of Experiment 1. To rule out the possibility that such variations in recall performance in the study-only condition contributed to the pattern of results observed in Experiments 2a and 2b, we again conducted an additional ANOVA which included only the pooled data of the pretest conditions of Experiments 2a and 2b (see Fig. 3).

ANOVA revealed a significant main effect of LEVEL OF ATTENTION, *F*(1,102) = 89.19, MSE = 284.14, *p* < 0.001, *η*_*p*_^2^ = 0.47, reflecting overall higher recall rates for the full-attention condition than for the divided-attention condition (76.3% vs. 54.2%), but no main effect of POSITION OF SECONDARY TASK, *F*(1,102) = 1.82, MSE = 637.11, *p* = 0.18, *η*_*p*_^2^ = 0.02. Most important, there was again a significant interaction between factors, *F*(1,102) = 17.32, MSE = 284.14, *p* < 0.001*, η*_*p*_^2^ = 0.15. Specifically, while recall was impaired under divided attention, relative to full attention, both when the secondary task occurred in Stage 1 and in Stage 2, all *p*s < 0.001, this difference between the divided-attention and full-attention conditions was greater when the secondary task took place during Stage 2 than during Stage 1 (31.8% vs. 12.3%). The findings of the additional analysis thus converge with the findings from the earlier analyses that included the study-only condition.

### Discussion

As expected, the material-specific secondary task used in Experiment 2 induced higher levels of interference than the material-general secondary task used in Experiment 1: compared to the full-attention condition, final-test recall in the divided-attention condition was overall more impaired for study-only (baseline) pairs of Experiment 2 than of Experiment 1 (19.7% vs. 12.1%). Otherwise, the results of Experiment 2 replicate the findings of Experiment 1. In particular, the results of Experiment 2 showed the typical pretesting effect under full attention, which was eliminated when attention was divided during Stage 2 (Experiment 2b) but was increased when attention was divided during Stage 1 (Experiment 2a). Like in Experiment 1, this pattern of results persisted also when the analysis was limited to trials in which all responses were correct on the secondary task, suggesting that the increased pretesting effect under divided attention in Experiment 2a was not merely a result of strategic allocation of attentional resources.

Furthermore, pretesting once again reduced only the number of intrusions produced on the final test when the secondary task was applied during Stage 1, but not during Stage 2. Finally, like in Experiment 1, an additional analysis including only the pooled data of Experiments 2a and 2b of the pretest condition showed that compared to learning under full attention, correct recall on the final test was impaired more when the secondary task occurred during Stage 2 than during Stage 1. The results thus suggest once again that primarily the attentional processes operating during Stage 2 of the learning trial are critical for the pretesting effect.

## General discussion

The present experiments are the first in the literature to investigate the influence of divided attention on the pretesting effect. The results suggest that both the initial pretest stage (Stage 1) and the subsequent feedback stage (Stage 2) of the learning trial may require attentional resources, with Stage 2 being particularly demanding on attentional resources. Specifically, results first of all showed a typical pretesting effect when learning occurred under full attention, as reflected in greater final-test recall of pretested pairs than study-only pairs. Under divided attention, the pretesting effect was even increased in size when the secondary task took place during Stage 1 of the learning trial but was eliminated when the secondary task occurred during Stage 2 of the learning trial. The results of additional analyses which included only the pretest trial data expanded upon these findings, by showing that final-test recall of pretest pairs was impaired both when distraction occurred during Stage 1 and Stage 2, but with a more pronounced impairment effect when it occurred during Stage 2. This pattern of results held regardless of whether the secondary task induced material-general interference (Experiment 1) or material-specific interference (Experiment 2).

### Theoretical impact

The present finding that distraction during Stage 2, but not Stage 1, of the learning trial can eliminate the pretesting effect indicates that processes which occur during Stage 2 and require attentional resources contribute to the pretesting effect. This conclusion fits well with the attentional account of the pretesting effect which suggests that Stage 2 is critical for the effect since pretesting is assumed to increase subjects’ curiosity to learn the correct response, thus boosting attentional encoding of the subsequent feedback in Stage 2, resulting in enhanced recall of pretested material. Applying a secondary task during Stage 2 should therefore limit participants’ capacity to encode the target word more thoroughly after a pretest and thus reduce the size of the pretesting effect, which is exactly what the results of the present Experiments 1 and 2 demonstrate.

At first glance, however, the attentional account does not provide an explanation for the finding of our additional analyses that distraction during Stage 1 can cause a small, but reliable memory impairment for pretested pairs on the final test. Indeed, this finding indicates that attention-requiring processes also operate during Stage 1. One possible explanation arises from a prior study which showed that participants’ attention levels increase under full attention *after* they have made their guess (Potts et al., 2019). Since it takes participants a few seconds on average to come up with a guess, this attentional increase should typically occur only within the last few seconds of Stage 1. When attention is divided during Stage 1, such pretest-induced increases in attention levels might be limited—at least when the guess is made relatively early in Stage 1—and thus prevent, to some degree, a more effective encoding of the correct response in Stage 2. As a result, the observed (slight) final-test recall impairment under divided, relative to full, attention might occur for pretested information.

Interestingly, a recent study provides further evidence that attentional processes can play a critical role for the pretesting effect. In this study, Sana and Carpenter (2023) found that answering pretest questions about a subsequently studied prose passage (Experiment 1) or video lecture (Experiment 2) only enhanced final-test recall performance of initially *untested* details when the answers to the pretest questions were found in the second half of the passage, but not the first half. To explain these findings, the researchers proposed the attentional-window hypothesis, which assumes that pretesting boosts participants’ attentional levels until the correct answer to the question has been found. This account can explain the pattern of results because final-test recall of initially untested information should get boosted when the information appears ‘in the attentional window’, i.e., prior to the tested information, but not when the information appears after the tested information. This prior study, together with the present study, thus emphasizes the potential role of attentional processes for the pretesting effect for a variety of study materials, i.e., paired associates, prose passages, and video segments.

The question arises whether other prominent hypotheses of the pretesting effect, such as the elaboration account or the search-set account, might also be able to explain the present pattern of results. The elaboration account assumes that pretesting during Stage 1 leads to the activation of memory representations related to the cue word, which are integrated with the cue-target pair once feedback is provided in Stage 2 and can be used as semantic mediators on the final test through which the target word is retrieved (Cyr & Anderson, 2012, 2018; Huelser & Metcalfe, 2012). Critically, however, the literature is divided on whether elaborative processing requires attentional resources, with some researchers arguing that this type of processing is mostly automatic (e.g., Anderson, 1983) and some proposing that it is effortful and vulnerable to distraction (e.g., Craik et al., 1996; Mulligan, 2008). Consequently, it is not possible to come to an unequivocal conclusion about whether or not the present findings fit with the elaboration account.

The search-set account assumes that the attempt to guess the target word when provided with a cue word on the pretest in Stage 1 should activate a search set consisting of items related to the cue word, including the correct answer, and thus lead to a more efficient encoding of the target word when corrective feedback is given in Stage 2 (e.g., Grimaldi & Karpicke, 2012). Similar to the elaboration account, it has not been specified whether the pretest-induced activation of the search set should require attentional resources, which makes it difficult to assess whether the present findings align or contradict this particular account. It should therefore be a high priority for future research to develop versions of these two explanations that clarify whether, and to what extent, attentional processes might be involved.

### Relation to prior work

Our findings parallel observations from prior work examining the effects of divided attention on the testing effect. In particular, Buchin and Mulligan (2017, Experiments 2 and 3) applied a typical testing-effect task in which, during Stage 1 of the learning trial, participants first studied word pairs (e.g., star—*night*), before, in Stage 2 of the learning trial, they either restudied the information or performed a posttest in which the target word had to be retrieved from the cue word (e.g., star—*?*). While results replicated the typical testing effect under full attention, with greater recall on a subsequent final test of initially tested than restudied pairs, the testing effect was even increased in size when a secondary task was applied during Stage 2 of the learning trial (for similar results, see Mulligan & Picklesimer, 2016; Buchin & Mulligan, 2019). This pattern of results arose regardless of whether material-general or material-specific interference was induced.

The current Experiments 1a and 2a thus add to the parallels between the posttesting and the pretesting procedures that have been observed in earlier work. For instance, both the posttesting and the pretesting effect have been shown to increase with the length of the retention interval since both types of testing can reduce time-dependent forgetting (e.g., Kliegl et al., 2024a; Roediger & Karpicke, 2006). Furthermore, both types of testing can protect the tested material from interference-induced forgetting which often occurs when related material is encountered before final testing (Halamish & Bjork, 2011; Kliegl et al., 2023). Finally, both posttesting and pretesting have been found to be particularly beneficial for final-test performance when multiple initial test cycles are applied (Karpicke & Roediger, 2007; Kliegl et al., 2024b).

Very recently, Mulligan and Buchin (2024) also examined the effects of divided attention on the pretesting effect. In contrast to the present study, these researchers divided attention during the entire pretest and study-only trials—i.e., during both Stage 1 and Stage 2 of a trial—and found that the pretesting effect persisted under this type of distraction. In numerical terms, the magnitude of the pretesting effect was larger even under divided attention than under full attention, which is similar to the results of the present Experiments 1a and 2a. However, the Mulligan and Buchin findings appear to be inconsistent with the observations of the present Experiments 1b and 2b. Indeed, their finding that the pretesting effect persisted under distraction when it was applied during a full trial seems to indicate that attentional processes do not play a major role during Stage 2 of the trial either. The cause for this apparent discrepancy in findings is currently unclear. This holds while testing format may have influenced results. Indeed, while in the present study a cued recall format was employed, Mulligan and Buchin used an item recognition format, and test format has sometimes been found to influence the results of pretesting experiments (e.g., Potts & Shanks, 2014; Potts et al., 2019; Seabrooke et al., 2021).

On a more general level, however, the present findings align with numerous prior findings showing that divided attention has more detrimental effects on subsequent recall performance when it is applied during memory encoding than during memory retrieval (for a review, see Mulligan, 2008). Specifically, visual inspection of Fig. 2 suggests that, when compared to the full-attention condition, final-test recall in the divided-attention condition is more impaired for i) study-only than pretest trials when attention was divided during Stage 1 (Fig. 2a, c) and ii) pretest than study-only trials when attention was divided during Stage 2 (Fig. 2b, d). The first finding clearly fits with prior work showing that divided attention can impair encoding processes more than retrieval processes. The second finding also aligns with the prior work since, during Stage 2, participants were encoding the target word in the pretest trial for the first time, whereas they had already encoded the target word in the study-only trial for 6 s prior. Consequently, the divided-attention manipulation in Stage 2 should have minimal impact for final-test performance for study-only trials, which is exactly what our findings suggest (Fig. 3).

The demonstration of prior studies that, unlike divided attention during memory encoding, divided attention during memory retrieval can still trigger beneficial effects for later retention has another implication for the interpretation of our results: the finding of the present Experiments 1a and 2a that dividing attention during guessing (Stage 1) does not reduce the size of the pretesting effect indeed leaves room for the possibility that beneficial processes which are *not* affected by distraction occur during Stage 1. For instance, guessing during Stage 1 may induce elaboration processes which potentially do not require substantial attentional resources, but which nevertheless facilitate the retrieval of the target information on the final test, thus contributing to the pretesting effect (see above). Prior studies indeed have found some evidence for a critical role of such elaboration processes for the pretesting effect (e.g., Bartl et al., 2024). Thus, a central goal of future research should be to determine whether and how processes occurring during Stage 1 interact with processes occurring during Stage 2 to induce the pretesting effect.

## Conclusion

The results of the present two experiments show that divided attention during learning eliminates the pretesting effect only when the secondary task is applied during Stage 2 of the learning trial, but not when subjects engage in the task during Stage 1 of the learning trial. Consistently, the results of two additional analyses that included only pretest trials revealed that final-test memory showed greater impairments when the secondary task was applied during Stage 2 than Stage 1, although such impairment was present in both situations. Overall, the findings suggest that the processes occurring during Stage 2 of the learning trial require more attentional resources than those taking place during Stage 1. This conclusion largely fits with attentional accounts of the pretesting effect.

## Data Availability

All study materials and data have been made publicly available on the Open Science Framework and can be found at https://osf.io/nxdzv/. None of the reported experiments have been preregistered.

## Footnotes

1 Participants who did not respond 100% correctly in any of the pretest or restudy trials in the secondary task were removed from the data set. As a result, one participant was removed from each of Experiments 1a, 1b, and 2a, and four participants were removed from Experiment 2b.
